# Symptoms and signs of conjunctivitis as predictors of disease course in COVID-19 syndrome

**DOI:** 10.1186/s12348-021-00264-0

**Published:** 2021-09-22

**Authors:** Martina Ranzenigo, Elena Bruzzesi, Laura Galli, Antonella Castagna, Giulio Ferrari

**Affiliations:** 1 Infectious Diseases, IRCCS San Raffaele Scientific Institute, Milan, Italy; 2 Vita-Salute San Raffaele University, Milan, Italy; 3 Cornea and Ocular Surface Disease Unit, Eye Repair Lab, IRCCS San Raffaele Scientific Institute, Via Olgettina, 60, 20132 Milan, Italy

**Keywords:** COVID-19, Conjunctivitis, Ocular symptoms

## Abstract

**Background:**

Severe acute respiratory syndrome coronavirus 2 (SARS-CoV-2) can induce conjunctivitis signs and symptoms. However, limited information is available on their impact on COVID-19 disease phenotype. Quantification of ocular signs/symptoms can provide a rapid, non-invasive proxy for predicting clinical phenotype. Moreover, the existence and entity of conjunctival viral shedding is still debated. This has relevant implications to manage disease spread.

The purpose of this study was to investigate conjunctivitis signs and symptoms and their correlation with clinical parameters, conjunctival viral shedding in patients with COVID-19.

**Methods:**

Fifty-three patients hospitalized between February 25th and September 16th, 2020 at the San Raffaele Hospital, in Milan, Lombardy, Italy with a confirmed diagnosis of SARS-CoV-2 were evaluated. Presence of interstitial pneumonia was confirmed with computed tomography scan imaging. Ocular signs and symptoms, anosmia/ageusia, clinical/laboratory parameters, and reverse transcriptase–polymerase chain reaction (RT-PCR) from nasopharyngeal and conjunctival swabs for COVID-19 virus were analyzed.

**Results:**

Forty-six out of 53 patients showed a positive nasopharyngeal swab for SARS-CoV-2 infection at the time of conjunctival evaluation. All the conjunctival swabs were negative. Conjunctivitis symptoms were present in 37% of patients. Physician-assessed ocular signs were detected in 28% of patients.

Patients with ocular symptoms or signs tended to be older: 76.8 years (62.4–83.3) vs 57.2 years (48.1–74.0), *p* = 0.062 and had a longer hospitalization: 38 days (18–49) vs. 14 days (11–21), *p* = 0.005. Plasma levels of Interleukin-6 were higher in patients with signs or symptoms in comparison with those without them: 43.5 pg/ml (19.7–49.4) vs. 8 pg/ml (3.6–20.7), *p* = 0.02. Red cell distribution width was also significantly higher: 15 (14.3–16.7) vs 13.2 (12.4–14.4), *p* = 0.001.

**Conclusions:**

We found that over a third of the patients had ocular signs or symptoms. These had higher prevalence in patients with a more severe infection. No viral shedding was detected in the conjunctiva. Our results suggest that prompt detection of conjunctivitis signs/symptoms can serve as a helpful proxy to predict COVID-19 clinical phenotype.

## Background

Conjunctivitis is probably the most common [1]ocular manifestation of COVID syndrome, and specific signs and symptoms have been frequently reported [2–4]. However, it is still unclear whether ocular involvement is associated with viral shedding in the conjunctiva/tears or it is rather a secondary involvement induced by the systemic infection. In this study, we investigated the prevalence of patient-reported ocular symptoms, physician-detected ocular signs, together with anosmia/ageusia and conjunctival viral shedding in a cohort of patients affected by COVID-19 infection.

## Materials and methods

We evaluated 53 patients hospitalized between February 25th and September 16th, 2020 at the San Raffaele Hospital, in Milan, Italy with a confirmed diagnosis of COVID-19 infection. Presence of interstitial pneumonia was confirmed in all 53 patients with computed tomography scan imaging [5]. This study did not include critically ill (i.e. intubated) patients. Paired (within 3 days) nasopharyngeal and conjunctival swabs were performed during hospitalization. The conjunctival samples were collected from both eyes using a single swab, which was then stored in a vial and analysed within 24 h. Swab specimens were processed by Cobas® SARS-CoV-2 Test (Roche), which detects ORF-1a/b and E gene regions on SARS-CoV-2 genome, designed to be used on the automated Cobas® 6800 Systems [6]. We also reported the cycle threshold (Ct) values, when available, which are a useful proxy of viral load [7].

A 4–item questionnaire was administered to patients in order to investigate symptoms of conjunctivitis (red eyes, sticky eyes, tearing, burning) and presence of ageusia or anosmia. Finally, patients were evaluated by a study physician for the presence of the following ocular signs: conjunctival hyperaemia, secretion, chemosis and epiphora; previous history of ocular diseases was also collected.

The study was conducted in accordance with the Declaration of Helsinki [8] and the evaluated patients are part of the COVID-19 institutional clinical-biological cohort (Covid-BioB registered on the ClinicalTrials.gov website: NCT04318366), whose study protocol was approved by the Hospital Ethics Committee (protocol number 34/int/2020). Informed consent was obtained according to the Ethic Committee guidelines. Patients’ demographic and clinical characteristics, treatment, need/mode of oxygen support as well as values of laboratory parameters during hospitalization were extracted from the Covid-BioB database.

### Statistical analysis

Median values and quartiles (IQR), were used to describe continuous variables while frequencies and percentages were used for categorical variables. Prevalence of patients with ocular symptoms or signs or ageusia or anosmia in the overall sample were estimated with the corresponding 95% confidence intervals (95% CIs) using the modified Wald method [9]. Characteristics of patients with or without symptoms or signs were compared using the Chi-square or Fisher’s exact test for categorical variables, and the Wilcoxon rank sum test for continuous variables.

Multivariate general linear regression models were fit to estimate mean differences in continuous outcomes (laboratory parameters at conjunctival swab) comparing patients with vs without ocular symptoms or signs, adjusted for two potential confounders [use of steroids before or at ocular swab (yes vs no) and timing of ocular swab execution since hospitalization (continuous variable in days)].

Two-tailed *P* values are reported and a *P* < 0.05 considered to indicate statistical significance.

Statistical analyses were performed with the SAS Software, release 9.4 (SAS Institute, Cary, NC).

## Results

Forty-six out of 53 patients showed a positive nasopharyngeal swab for SARS-CoV-2 infection at the time of conjunctival evaluation and were considered for further analysis. The median age was 65.0 years (48.1–78.0) and 24 (52%) were males. Four patients had a history of previous ocular diseases: 2 patients reported dry eye disease, 1 glaucoma and 1 ectropion.

All the conjunctival swabs were negative. Ocular symptoms were present in 17 [37%, 95% confidence interval (95%CI): 24.5–51.4] patients. Red eye was reported by 3 (7%) patients, sticky eyes by 4 (10%), tearing by 6 (15%), burning by 7 (16%). Patients’ characteristics according to the presence of ocular symptoms are reported in Table 1.

**Table 1:** Patients’ characteristics according to the presence of ocular symptoms

Variable	Overall (*n* = 46)	With ocular symptoms or signs (*n* = 17)	Without ocular symptoms or signs (*n* = 29)	*p*-value^§^
**Demographic and clinical characteristics**				
Age (years)	65.0 (48.1–78.0)	76.8 (62.4–83.3)	57.2 (48.1–74.0)	0.062
Male gender	24 (52.2%)	10 (58.8%)	14 (48.3%)	0.552
Days of symptoms before hospitalization	6 (3–11)	6 (2.5–10.5)	6 (4–11)	0.858
Days to conjunctival swab since hospitalization	4 (1–15)	11 (4–22)	3 (1–6)	**0.023**
Days of hospitalization	18 (12–38)	38 (18–49)	14 (11–21)	**0.005**
Reported hyperemia	3 (7%)	3 (21.4%)	0 (0%)	–
Sticky eyes	4 (10%)	4 (28.6%)	0 (0%)	–
Reported tearing	6 (15%)	6 (42.9%)	0 (0%)	–
Burning	7 (16.3%)	7 (50%)	0 (0%)	–
Hyperemia	3 (7%)	3 (17.6%)	0 (0%)	–
Tearing	9 (19.6%)	9 (52.9%)	0 (0%)	–
Secretion	6 (13%)	6 (35.3%)	0 (0%)	–
Reported chemosis	0 (0%)	0 (0%)	0 (0%)	–
Reported anosmia	11 (23.9%)	4 (23.5%)	7 (24.1%)	0.999
Reported ageusia	13 (28.3%)	3 (17.6%)	10 (34.5%)	0.315
Antiviral therapy (≥1 drug)	23 (50%)	11 (64.7%)	12 (41.4%)	0.221
Hydroxychloroquine	10 (21.7%)	6 (35.3%)	4 (13.8%)	0.139
Lopinavir	1 (2.2%)	1 (5.9%)	0 (0%)	0.370
Remdesivir	16 (34.8%)	7 (41.2%)	9 (31%)	0.534
Azithromycin	3 (6.5%)	2 (11.8%)	1 (3.4%)	0.545
Corticosteroids	22 (47.8%)	8 (47.1%)	14 (48.3%)	0.999
Dexamethasone	12 (26.7%)	3 (17.6%)	9 (32.1%)	0.488
Methylprednisolone	9 (20%)	5 (29.4%)	4 (14.3%)	0.265
Prednisolone	1 (2.2%)	0 (0%)	1 (3.6%)	0.999
CPAP	6 (13%)	1 (5.9%)	5 (17.2%)	0.390
Venturi mask	19 (41.3%)	6 (35.3%)	13 (44.8%)	0.555
Glasses	12 (26.1%)	6 (35.3%)	6 (20.7%)	0.314
Use of CPAP/Venturi mask/glasses	29 (63.0%)	11 (64.7%)	18 (62.1%)	0.998
Eyepiece diseases	4 (8.7%)	2 (11.8%)	2 (6.9%)	0.619
***Laboratory parameters***				
Ferritine at hospitalization (ng/mL)	534 (359–1209.5)	495 (158–1966)	573 (363–1106)	0.999
Ferritine at conjunctival swab (ng/mL)	390 (252–663)	663 (158–714)	385 (287–520.5)	0.958
Fibrinogen at hospitalization (mg/dL)	511 (447–602)	496.5 (432–698)	512 (457–598)	0.983
Fibrinogen at conjunctival swab (mg/dL)	469 (415–578)	494 (432–592)	461.5 (410–544)	0.615
Interleukin-6 at hospitalization (pg/mL)	21.7 (9.8–32.3)	16.2 (9.9–51.4)	22.25 (6.4–31.5)	0.412
Interleukin-6 at conjunctival swab (pg/mL)	14.9 (5.4–40.6)	43.5 (19.7–49.4)	8 (3.6–20.7)	**0.016**
Lactate dehydrogenase at hospitalization (U/L)	272 (230–322)	295 (230–332)	260 (229–321.5)	0.399
Lactate dehydrogenase at conjunctival swab (U/L)	256 (215–287)	256 (217–311)	255.5 (210–278.5)	0.817
Total lymphocytes at hospitalization (10^9^cells/L)	1 (0.7–1.3)	0.9 (0.8–1.1)	1.1 (0.7–1.3)	0.493
Total lymphocytes at conjunctival swab (10^9^cells/L)	1.2 (0.8–1.6)	1.3 (1–1.6)	1 (0.8–1.5)	0.438
Total monocytes at hospitalization (10^9^cells/L)	0.45 (0.3–0.7)	0.5 (0.3–0.7)	0.4 (0.3–0.6)	0.527
Total monocytes at conjunctival swab (10^9^cells/L)	0.5 (0.4–0.7)	0.5 (0.5–0.8)	0.4 (0.3–0.7)	0.081
Total neutrophils at hospitalization (10^9^cells/L)	4.2 (2.5–6.2)	5.2 (3.4–9.5)	4 (2.4–5.7)	0.227
Total neutrophils at conjunctival swab (10^9^cells/L)	4.55 (2.5–5.6)	5 (4–5.6)	4 (2.3–5.5)	0.158
PCR at hospitalization (mg/L)	39.05 (19–72.1)	65.1 (30.4–80.9)	31.1 (14–62)	**0.041**
PCR at conjunctival swab (mg/L)	18 (5.4–38.4)	29.6 (10.6–38.4)	11.05 (3.45–36.2)	0.228
PCT at hospitalization (ng/mL)	0.91 (0.35–2.06)	0.67 (0.35–2.06)	1.14 (0.56–1.61)	0.999
PCT at conjunctival swab (ng/mL)	0.65 (0.39–1.01)	0.95 (0.43–1.14)	0.64 (0.35–0.85)	0.368
Platelet at hospitalization (10^9^/L)	188.5 (150–225)	204 (169–225)	176 (147–215)	0.195
Platelet at conjunctival swab (10^9^/L)	193 (159–258)	191 (171–258)	195 (154–254)	0.741
RDW at hospitalization (%)	13.9 (12.7–15.3)	15.1 (13.7–16.5)	13.2 (12.5–14.4)	**0.012**
RDW at conjunctival swab (%)	14.1 (12.6–15.5)	15 (14.3–16.7)	13.2 (12.4–14.4)	**0.001**
White blood cells at hospitalization (10^9^cells/L)	5.85 (4.1–7.7)	6.5 (4.7–10.4)	5.5 (3.8–7.3)	0.274
White blood cells at conjunctival swab (10^9^cells/L)	6.2 (4.3–7.9)	7.3 (5.5–8)	5.3 (4.1–7.7)	0.106
D-Dimer at hospitalization (μg/mL)	0.64 (0.43–1.27)	0.65 (0.59–1.53)	0.6 (0.38–1.01)	0.235
D-Dimer at conjunctival swab (μg/mL)	0.4 (0.33–0.63)	0.58 (0.34–0.71)	0.38 (0.27–0.55)	0.165
PaO_2_/FiO_2_ at hospitalization (%) (*n* = 28)	3.04 (2.05–3.36)	2.83 (1.77–3.32)	3.22 (2.07–3.42)	0.416
PaO_2_/FiO_2_ at conjunctival swab (%) (*n* = 14)	2.36 (1.61–3.18)	2.04 (1.84–2.34)	2.75 (1.41–3.4)	0.533
SaO_2_ at hospitalization (%) (*n* = 40)	94.55 (92.4–96.15)	94.4 (91.8–96.3)	94.9 (92.4–96)	0.459
SaO_2_ at conjunctival swab (%) (*n* = 23)	95.7 (94.2–97.3)	97.4 (96.3–99.3)	95.3 (94.2–96)	0.069
Uric acid at hospitalization (mg/dL)	4.4 (3.65–6.35)	3.9 (3.8–6.3)	4.5 (3.6–6.6)	0.630
Uric acid at conjunctival swab (mg/dL)	4.3 (3.7–6)	3.9 (3.8–5.2)	4.65 (3.5–6.2)	0.467
Urea at hospitalization (mg/dL)	32 (23.5–42.5)	33 (29–49)	31 (23–40)	0.254
Urea at conjunctival swab (mg/dL)	31 (24–46)	39 (31–49)	27 (22–40)	0.189
ALT at hospitalization (U/L)	31.5 (18–39.5)	31 (15–36)	32 (21–41)	0.244
ALT at conjunctival swab (U/L)	33.5 (20–52.5)	32.5 (22–52.5)	35 (18.5–55)	0.678
AST at hospitalization (U/L)	27 (23–36)	33 (21–46)	26 (23.5–32)	0.331
AST at conjunctival swab (U/L)	27 (22–36)	27 (23.5–34.5)	27 (21–47)	0.742
CK at hospitalization (U/L)	65.5 (46–140)	131 (37–249)	62 (46–100)	0.227
CK at conjunctival swab (U/L)	43 (27–100)	27 (22–49)	56 (35.5–100)	0.056
Creatinine at hospitalization (mg/dL)	0.96 (0.77–1.1)	0.95 (0.77–1.28)	0.96 (0.77–1.07)	0.594
Creatinine at conjunctival swab (mg/dL)	0.83 (0.73–1.01)	0.88 (0.79–1)	0.78 (0.72–1.03)	0.400
Nasopharyngeal swab Ct at conjunctival swab	30.38 (25.05–32.75)	29.5 (24.34–32.75)	32.51 (25.05–35.01)	0.432

Abbreviations: PCR, polymerase chain reaction; PCT, procalcitonin; RDW, red cell distribution width; ALT, alanine transaminase; AST, aspartate aminotransferase; CK, creatine kinase

Results are described by median (IQR) or frequency (%), as appropriate

§ by chi-square/Fisher’s exact test (categorical variables) or Wilcoxon rank-sum test (continuous variables)

The study physician observed ocular signs in 13 [28%, 95% confidence interval (95%CI): 17.2–42.7] patients: epiphora in 9 (20%) patients, secretion in 6 (13%), conjunctival hyperaemia in 3 (7%), chemosis in 0%; anosmia was reported in 11 (24%, 95% confidence interval (95%CI): 13.8–38.1] patients and ageusia in 13 [28%, 95% confidence interval (95%CI): 17.2–42.7].

Patients with ocular symptoms or signs tended to be older: 76.8 years (62.4–83.3) vs 57.2 years (48.1–74.0), *p* = 0.062 and had a longer hospitalization: 38 days (18–49) vs. 14 days (11–21), *p* = 0.005 (Table 1).

Importantly, at swab sampling, Ct values were similar between patients with signs or symptoms than those without them [29.5 (24.3–32.8) vs 32.5 (25.1–35.0), *p* = 0.432].

Plasma concentrations of Interleukin-6 were significantly increased in patients with signs or symptoms in comparison with those without them: 43.5 pg/ml (19.7–49.4) vs. 8 pg/ml (3.6–20.7), *p* = 0.02. Red cell distribution width was also significantly higher: 15 (14.3–16.7) vs 13.2 (12.4–14.4), *p* = 0.001. Finally, oxygen saturation, PaO2/FiO2 ratio or the number of patients treated with nasal cannula or Venturi mask or non-invasive mechanical ventilation were not affected by the presence of ocular signs or symptoms. Detailed information about patients presenting ocular signs or symptoms is reported in Table 2.

**Table 2:** Patients presenting ocular signs or symptoms

Subject	Age (years)	Gender	Sings	Symptoms	Anosmia	Ageusia	CPAP during hospitalization	Venturi mask during hospitalization	Glasses during hospitalization	Ct of nasopharyngeal swab at conjunctival swab	Nasopharyngeal swab at conjunctival swab	Days to conjunctival swab since hospitalization	Days of hospitalization	PaO_2_/FiO_2_ at hospitalization	Oxygen saturation (%) at hospitalization
1	81	Female	Hyperemia, tearing, secretion	Hyperemia, tearing, sticky eyes, burning	No	No	No	No	Yes	–	Positive	11	32	3.13	94.8
2	89	Female	Secretion		No	No	No	No	No	–	Positive	5	49	5.35	94.5
3	65	Male		tearing	No	Yes	No	Yes	No	25.48	Positive	5	19	3.38	94.0
4	80	Male	Hyperemia	Hyperemia, sticky eyes, burning	Yes	Yes	No	No	Yes	22.1	Positive	17	34	3.30	95.0
5	72	Female	Secretion		No	No	Yes	Yes	No	24.34	Positive	5	18	2.71	91.8
6	62	Male	Tearing		No	No	No	No	Yes	–	Positive	23	76	–	–
7	93	Male	Tearing	Tearing, sticky eyes	No	No	No	Yes	Yes	–	Positive	37	50	0.99	88.6
8	77	Male	Tearing	Tearing, burning	Yes	No	No	No	No	30.97	Positive	56	63	0.99	97.1
9	77	Male	Tearing, secretion		No	No	No	No	No	–	Positive	22	41	–	–
10	38	Male	Hyperemia		No	No	No	No	No	33.04	Positive	33	42	–	24.4
11	48	Female		Burning	No	No	No	No	Yes	32.49	Positive	1	12	–	96.3
12	83	Female	Tearing, secretion		No	No	No	No	No	39.47	Positive	4	38	1.49	98.3
13	46	Male		Burning	Yes	No	No	Yes	No	–	Negative	4	15	2.94	94.4
14	31	Female	Tearing	Burning	Yes	Yes	No	No	No	17.68	Positive	0	9	–	97.5
15	77	Male		Hyperemia, tearing	No	No	No	Yes	No	20.4	Positive	1	4	2.04	92.7
16	89	Female	Tearing		No	No	No	No	Yes	32.75	Positive	20	41	3.33	92.7
17	94	Male	Tearing, secretion	Tearing, sticky eyes, burning	No	No	No	Yes	No	25.07	Positive	15	49	2.45	90.2

At multivariate analysis, plasma concentrations of Interleukin-6 and red cell distribution width were on average higher in patients with signs or symptoms (IL-6 β: + 32.6, 95%CI: 1.33–64.0, *p* = 0.042; RDW β: + 2.15, 95%CI: 0.91–3.39, *p* = 0.001) compared to patients without them after adjusting for confounders; a marginally significant difference was seen in Ct values suggesting lower values (i.e. higher viral load) for patients with signs or symptoms when adjusting for confounders (Ct β: -4.32, 95% CI: − 9.30, 0.66, *p* = 0.086).

## Discussion

In this paper, we show that conjunctivitis symptoms/signs are associated with COVID-19 disease course. This is supported by a number of observations. First, the finding of elevated levels of IL-6, an important predictor of disease severity [10] [11]. Second, patients with ocular manifestations showed lower Ct values (i.e. higher viral load) in rhinopharyngeal swabs. Third, we also found that patients with ocular symptoms had a longer hospitalization, suggesting a relationship between ocular symptoms and severity of the disease. However, no significant difference in patients’ age -one of the most important prognostic factor- was found between the two groups. Similarly, no significant difference was detected in the type of therapies administered during the hospital stay, which could have had an impact on viral clearance. Importantly, ocular signs/symptoms were not induced by ventilatory support, as our analysis failed to detect any association between the two.

Therefore, our study suggests that testing for ocular symptoms/signs could be a helpful tool to alert clinicians on a more severe clinical phenotype. In this vein, our results are corroborated by findings from Ping Wu et al. [12]

We did not find expression of COVID-19 RNA on the conjunctiva of our patients, similarly to others, who repeatedly tested a cohort of COVID-19 patients [13]. Azzolini et al [14] recently found virus expression in the conjunctiva, although clinical and methodological differences make comparisons difficult. However, the role of the ocular mucosa as a potential entry site or as a potential reservoir for the virus remains unclear.

We acknowledge some limitations including the cross-sectional design of the study, the limited number of patients and the timing of swab collection that varied as a consequence of the critical situation induced by COVID-19 in our hospital.

Importantly, ocular signs/symptoms were not induced by ventilatory support, as our statistical analysis failed to detect any association between the two.

## Conclusion

In summary, we found that over a third of the patients had ocular signs or symptoms and about a quarter presented anosmia and ageusia. While additional studies are needed to confirm our findings, we propose that testing ocular signs and symptoms at hospitalization can be an effective, non-invasive and rapid screening measure of COVID-19 patients.

## Data Availability

The datasets used and/or analysed during the current study are available from the corresponding author on reasonable request.

## Abbreviations

SARS-CoV-2: Severe acute respiratory syndrome coronavirus 2
Ct: cycle threshold
CI: confidence interval
